# Particle Swarm Contour Search Algorithm

**DOI:** 10.3390/e22040407

**Published:** 2020-04-02

**Authors:** Dominik Weikert, Sebastian Mai, Sanaz Mostaghim

**Affiliations:** Faculty of Computer Science, Otto-von-Guericke University Magdeburg, 39106 Magdeburg, Germany; sebastian.mai@ovgu.de (S.M.); sanaz.mostaghim@ovgu.de (S.M.)

**Keywords:** particle swarm optimisation, contour search, flat landscape, multi-modal

## Abstract

In this article, we present a new algorithm called Particle Swarm Contour Search (PSCS)—a Particle Swarm Optimisation inspired algorithm to find object contours in 2D environments. Currently, most contour-finding algorithms are based on image processing and require a complete overview of the search space in which the contour is to be found. However, for real-world applications this would require a complete knowledge about the search space, which may not be always feasible or possible. The proposed algorithm removes this requirement and is only based on the local information of the particles to accurately identify a contour. Particles search for the contour of an object and then traverse alongside using their known information about positions in- and out-side of the object. Our experiments show that the proposed PSCS algorithm can deliver comparable results as the state-of-the-art.

## 1. Introduction

Finding the contour of an object, known as contour search, has a large variety of applications. Beside the conventional applications in image processing, there are other applications in robotics. Imagine a scenario of identifying the contour of wildfire or oil spill on the ocean. The major challenges concern searching for an object and then identifying the corresponding contour. The existing algorithms typically rely on image processing technologies [1,2,3], which typically require knowledge about the entire search space.

The goal of this paper is to propose a new algorithm for contour search which is based on Particle Swarm Optimization (PSO) [4]. We aim to unify both of the search processes for the object and the contour identification in one algorithm. Since we work on PSO for search, this algorithm can be easily applied to real-world applications such as in collective robotic search scenarios for the detection of wildfires and oil spills. In this paper, we aim to provide the algorithmic background for contour search. Applications in robotics require further specific refinements, which are out of the scope of this paper.

In the following, we consider the contour search of an object with an unknown shape which is located in an unknown position in a two-dimensional search space. Similar to optimization scenarios, the goal is to find and identify the contour (border) of any encountered part of the object. In this paper, we assume that the parts of the search space which do not contain the object can be modelled by a flat fitness landscape and therefore, it is very difficult for optimization algorithms to pursue the search. Furthermore, we assume that the area of the search space which includes the object, maps also to only one fitness value which is different than the other one. This implies that we have a search space which can only be mapped into two fitness values (i.e., we have black and white fitness **landscape). The ultimate goal is to find a set of solutions which represent a good approximation of the** contour. In the literature on optimization algorithms, such optimization problems can be modelled as multi-modal optimization problems [5] with the difference that we only want to identify the multi-modal solutions which are located at the border of an object. Similar to multi-modal problems, all of these multi-modal solutions map to the same fitness function.

While PSO has been shown to be a very efficient optimization algorithm for many optimization problems [6,7,8,9], where its performance relies on the existence of a non-zero gradient in a search space. In contour search, existence of a gradient can not be guaranteed, with a search space consisting of a flat fitness landscape of equal values and non-monotonic jumps from one fitness value to the other. This work aims to adapt the principles of Particle Swarm Optimisation and designs the movement of a swarm to find and follow a contour in a search space.

The article is structured as follows—Section 2 defines the problem of contour search for the context of this paper. Section 3 provides the related works for contour search in image processing. In Section 4, the principles of PSO are explained. Section 5 contains a detailed description of the proposed contour search algorithm. Section 6 includes the experiments, comparison and the evaluations of the proposed method. Section 7 concludes the paper and provides new directions for future work.

## 2. Problem Definition

**Figure 1:**
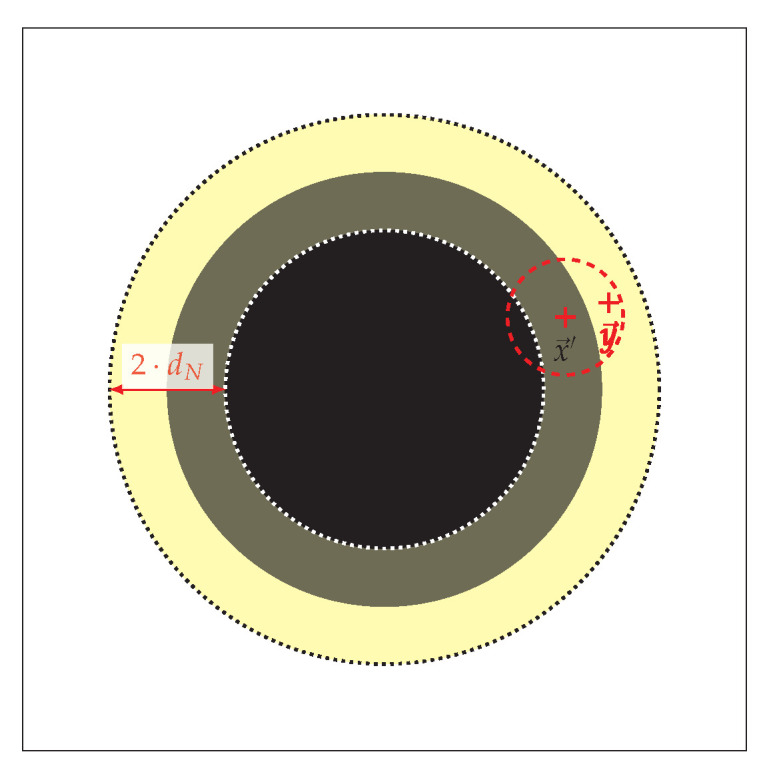
A black circle as an object in a search space. The highlighted area illustrates the set *P* representing the contour.

In order to deal with the contour search problem using a PSO approach, we define the problem as follows. We assume that we have a search space $S=R^{2}$ and all the solutions (points or pixels) in *S*, denoted by $\vec{x}\in S$ have a fitness value $f(\vec{x})$. Using this fitness value we can identify various objects in the search space. The fitness value can be binary such as black and white in a binary image. Furthermore, we assume that these solutions are concentrated in various regions in the search space representing objects. One object is shown as a binary image in Figure 1. Suppose we have a neighbourhood set for each solution $\vec{x}$ as $N\left(\vec{x}\right)=\left\{\forall \vec{y}\in S|Dist\left(\vec{x},\vec{y}\right)<d_{N}\right\}$, where Dist defines a distance measurement (L1 or L2 norms) and $d_{N}$ is a parameter defining the size of the neighbourhood. The goal of contour search is to identify a set *P* of the solutions located at the edges between these regions. In this case, *P* is the set of solutions ${\vec{x}}^{\prime }$ that have at least one neighbouring solution $\vec{y}$ with a different value of *f* as shown in Figure 1. We define: $P=\{\forall {\vec{x}}^{\prime }\in S|\exists \vec{y}\in N\left({\vec{x}}^{\prime }\right):f\left(\vec{y}\right)\neq f\left({\vec{x}}^{\prime }\right)\}$. This means that both objective values are contained in the surrounding fitness landscape and we assume that there is a sharp distinction for $d_{N}⟶0$. This implies that, for an image containing a black circle with radius *r* in a white background (as shown in Figure 1), the contour can be specified by *P* which is the region located between two circles with $r_{1}=r+d_{N}/2$ and $r_{2}=r-d_{N}/2$ and all three circles share the same center point. Ideally for $\lim_{d_{N}⟶0}$, *P* represents the set of solutions on the border (edge) of the circle.

In image processing, the search space is usually considered to be a discrete space that is represented by pixels. Therefore, these two neighbouring solutions ${\vec{x}}^{\prime }$ and $\vec{y}$ are usually two neighbouring pixels which different colours (*f* values). Since we work on continuous space in this paper, the neighbourhood is defined by random sampling in the surrounding area defined by the neighbourhood *N*. Hence, the outcome of our approach is an approximation of *P* with a limited size as described in Section 5.

## 3. Related Works

Contour search (also called edge detection) has been the subject of research for multiple decades, with most work being on image processing technologies. Such techniques require a complete overview of the search space and apply mathematical operators to every pixel of an image. Edges in image processing are usually defined as maxima of the gradient value of an image. These maxima represent large changes in pixel values and are indicative of object boundaries. Object contours are therefore continuous edges forming a closed loop, for example, they fulfil the additional constraint that their first and last points are identical. There are various ways to identify the edges in an image, which are mostly based on two main methods—Gradient based edge detection, which directly searches for gradient maxima, and laplacian based edge detection, which relies on detecting zero points in the second derivative of the image. Much research has been conducted into improving the performance of edge detection algorithms [10,11,12].

The underlying techniques mostly rely on kernel convolution, canny edge detection, adaptive contour models, or border following methods. Kernel convolution is used for effects such as blurring and sharpening as well as edge detection. A kernel is a convolution matrix, or mask, that is applied to every pixel of the image. During convolution, the pixel value is added with its neighbours according to the weight provided by the kernel. This sum is then normalised by the total sum of coefficients of the kernel as to prevent brightening or darkening of the image. This kernel convolution technique can be used to detect edges by using symmetrical kernels with opposing sides being assigned different signs and the axis of symmetry being assigned 0. When this kernel overlays an edge, the corresponding pixels will have significantly different magnitudes and thus the convolution will result in a high value.

The Canny edge detection was proposed by John Canny in 1986 [1]. This algorithm is based on noise filtering, calculating intensity gradient, edge thinning and hysteresis thresholding.

In adaptive contour models, it is assumed that the contours differ from edges since they are supposed to be continuous lines that signify an object boundary. In such algorithms, edge detection is combined with adaptive contour models [2] to detect and follow contours.

The border-following methods are usually applied to binary images. In this approach, a contour is traced by sequentially searching the direct (moore) neighbourhood of a pixel for a black pixel using a relative order, for example, starting clockwise from the pixel to the left of the current pixel. This search is then repeated with the found pixel, forming a chain of pixels until the contour is complete. An example of this is the algorithm described by Duda et al. in Reference [13]. The algorithm starts by tracing each pixel of the image, for example, column-by-column from the bottom row to the top row, until a black pixel is encountered. Then, the pixel-following algorithm follows these steps:

1. Set the start pixel $s_{1}$ and the current pixel *c* to be the encountered black pixel. Set the current direction *d* to “up”
2. Examine the neighbours of *c* in a clockwise order from the current direction. The first encountered black pixel is the next pixel on the contour. If $c=s_{1}$, the found pixel is saved as the second pixel $s_{2}$. If no point is found, the contour consists of a single pixel and the algorithm terminates.
3. Set the step direction from the current pixel to the new pixel as the new direction. If the direction is diagonal, it is set to a counterclockwise of this direction.
4. If the current point and the new point are identical to the starting point $s_{1}$ and the second point *s*, respectively, a complete contour is found. If not, repeat from step 2 with the current point and direction set to the new point and direction.

This algorithm can be expanded to find multiple contours with topological information [14]. An extended and more efficient pixel-following algorithm is proposed by Seo et al. [3]. Since both References [3,14] use the pixel-following approach for contour search, which has similarities with our proposed method, we will use this pixel-following approach for comparisons.

## 4. Particle Swarm Optimisation

The basis for our developed algorithm is a modified Particle Swarm Optimization (PSO) [4] algorithm. A typical PSO algorithm maintains a population of particles. In each iteration of PSO, the position of these particles is adjusted according to its own experience and that of the swarm move in the search space. Each particle *i* is assigned a position ${\vec{x}}_{i}\left(t\right)$ and a velocity ${\vec{v}}_{i}\left(t\right)$ at a given time step *t*. Additionally, the particle *i* possesses a memory of its own so far obtained best position, denoted by ${\vec{x}}_{bi}\left(t\right)$ (called local best) and the best position ever attained by the swarm, the so called global best, ${\vec{x}}_{g}\left(t\right)$. The difference between the position of the particle *i* and the global best, ${\vec{x}}_{g}\left(t\right)-{\vec{x}}_{i}\left(t\right)$ is called the social component ${\vec{v}}_{i_{s}}\left(t\right)$, while the difference in position and local best ${\vec{x}}_{bi}\left(t\right)-\vec{x_{i}}\left(t\right)$ is called the cognitive component ${\vec{v}}_{i_{c}}\left(t\right)$. Using these components, the position of each particle, and thus the entire swarm, is then adjusted in each time step *t* according to the following equations:

(1) $$\begin{array}{cc} {\vec{v}}_{i}\left(t+1\right) & =\omega {\vec{v}}_{i}\left(t\right)+c_{1}{\vec{r}}_{1}⊗{\vec{v}}_{i_{c}}\left(t\right)+c_{2}\vec{r_{2}}⊗{\vec{v}}_{i_{s}}\left(t\right) \end{array}$$

(2) $$\begin{array}{cc} {\vec{x}}_{i}\left(t+1\right) & ={\vec{x}}_{i}\left(t\right)+{\vec{v}}_{i}\left(t+1\right). \end{array}$$

The parameters $r_{1}$ and $r_{2}$ represent vectors of uniformly distributed random variables in the range of [0,1]. These are included to implement randomness in the direction of the velocity, increasing the diversity of the particle population and are combined with the individual velocity components via element-wise multiplication ⊗. Oldewage et al. demonstrated that including element-wise vector multiplication for the stochastic variables improved performance of the swarm over that of a simple scalar multiplication, especially in multi-dimensional problems [15]. ω is called inertia weight and is used to control the influence of previous speeds of the particle. The influence of the cognitive and social components are scaled by $c_{1}$ and $c_{2}$, respectively.

The PSO algorithm encounters a challenge when confronted with problems with flat fitness landscapes. In order to enforce exploration in such search spaces Quantum PSO [16], charged PSO (CPSO) [17] has been proposed.

## 5. Particle Swarm Contour Search (PSCS)

In the following, we propose Particle Swarm Contour Search with the goal of finding the contour of one or more objects in a search space. To overcome the inherent weaknesses of PSO in flat landscapes, the velocity update is modified to put less importance on the existence of a gradient. The PSCS algorithm consists of three distinct phases with different velocity update mechanisms—Object Search Phase, Contour Search Phase and Contour Trace Phase.

Initially, all particles conduct a global search using a modified CPSO approach [18] (*Object Search Phase*). Once a particle detects an object, a new sub-swarm is created from its neighbourhood to explore the contour of that object (*Contour Search Phase*). When a particle exploring an object detects that it is close to an edge, it enters a contour following phase (*Contour Trace Phase*).

Algorithm 1 shows a pseudo-code description of the initialisation and overall structure of the algorithm. More detailed descriptions are given for each phase individually in the following.

The input of the algorithm consists of the search space *S*, the population size PopSize, the object detection threshold θ and the minimum contour distance ε. During the initialisation the positions, velocities and states (${\vec{in}}_{i}$ and ${\vec{out}}_{i}$) for all the particles are set and in this way a global swarm $S_{g}$ is created.

**Algorithm1:** Baseline PSCS algorithm Input: *S*, PopSize, θ, ε, *A*, SubSwarmSize; Output: *A*; A=∅; SubSwarms=∅; 
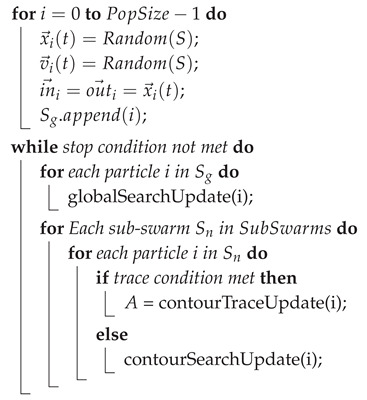


### 5.1. Initialisation

In this step, the particles and their variables are initialised with random positions and velocities to ensure non-biased exploration of the search space. With more knowledge of the search space or the object contour, specific patterns could be used to improve the exploration and the diversity.

In case of non-binary images, a threshold value θ for the detection of objects needs to be defined. This represents the minimum value for which the particle is considered to be inside an object. The particles are stored in a global swarm denoted by $S_{g}$. Additionally, we define two new parameters $\vec{in}$ and $\vec{out}$ for each particle *i*. These values are initialised to its current position.

### 5.2. Object Search Phase

In this phase, the velocities and the positions are updated for the global swarm $S_{g}$ including all particles that have not yet been assigned to a sub-swarm. The goal of this phase is to find the object(s) in the assigned search space and assign a sub-swarm to find the contour of each object. A pseudo-code algorithm is shown in Algorithm 2. Initially, this swarm will consist of all particles and eventually its size will be reduced to 0 as more sub-swarms are created to track object contours. Similar to the Charged-PSO approach [17], the particles in this swarm use the Equations (3) and (4) to update their velocities. Here, only the members in sub-swarms are being considered for the calculation of the repulsive force:

(3) $$\begin{array}{cc} {\vec{v}}_{i}\left(t+1\right) & ={\vec{v}}_{i}\left(t\right)+{\vec{a}}_{i}\left(t\right) \end{array}$$

(4) $$\begin{array}{cc} {\vec{a}}_{i}\left(t\right) & =\sum_{j\notin S_{g}}\frac{Q_{j}*Q_{j}}{\left(|{\vec{x}}_{i}\left(t\right)-{\vec{x}}_{j}\left(t\right)\right){)|}^{3}}\left({\vec{x}}_{i}\left(t\right)-{\vec{x}}_{j}\left(t\right)\right), \end{array}$$

where $Q_{i}$ and $Q_{j}$ are constants and define the intensity of repulsion. Equation (4) leads to a repulsive force acting on the global swarm from all sub-swarms, preventing the global swarm from repeatedly creating sub-swarms to explore the same object. This repulsion is decreased over time to allow all particles to trace the contour in case no other object is found.

When the fitness value of the particle *i* is below the given threshold θ (i.e., it has encountered an object to be explored), it creates a new sub-swarm and recruits SubSwarmSize−1 particles from its immediate neighbourhood. The corresponding value for $\vec{in}$ for all the members in the new sub-swarm is changed to the current position of particle *i*, that is, ${\vec{x}}_{i}$. If $f({\vec{x}}_{i})$ remains above θ, it simply updates its last known position outside any objects, denoted by $\vec{out}$. If any particle leaves the search space, it is simply reflected from the border of the search space with a small random additional change in direction. This reflective behaviour holds true for all phases. To prevent excessive particle speeds, particle velocity is clamped once it exceeds a maximum value depending on the search space size.

**Algorithm 2:** PSCS global search phase 

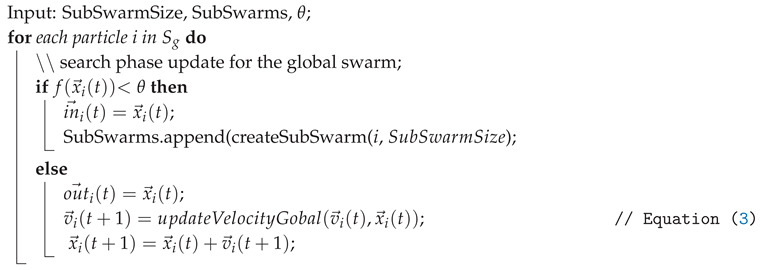



### 5.3. Contour Search Phase

Once a sub-swarm $S_{n}$ is created, it immediately enters the Contour Search Phase. The corresponding Algorithm in outlined in Algorithm 3.

**Algorithm 3:** PSCS contour seach and trace phase Input: ε,SubSwarms,θ; Output: *A*; 
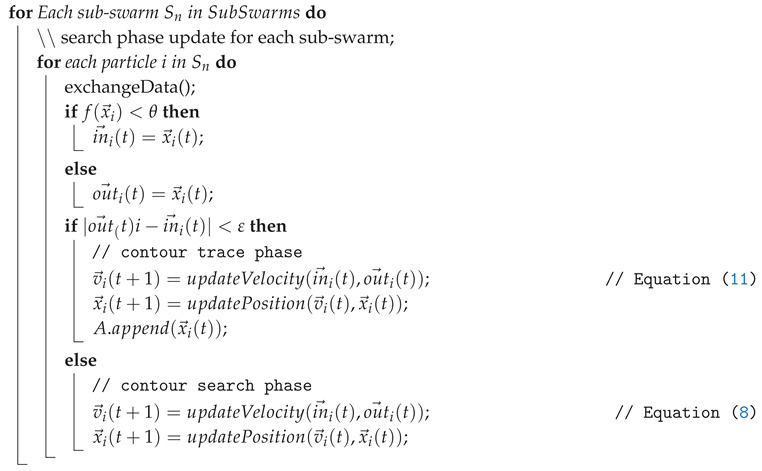


At the beginning of this phase, a data exchange is triggered between the current particle and a random particle of the same sub-swarm with a small probability. This leads to particles travelling through the contour and enables the algorithm to detect possible holes in the contour. Instead of tacking the local and global best positions, each particle updates either its last inside position ${\vec{in}}_{i}$ or outside position ${\vec{out}}_{i}$, depending on its fitness value. Accordingly, the velocity update is calculated as follows:

(5) $$\begin{array}{cc} \;{\vec{v}}_{i}^{in}\left(t\right) & ={\vec{in}}_{i}\left(t\right)-{\vec{x}}_{i}\left(t\right) \end{array}$$

(6) $$\begin{array}{cc} {\vec{v}}_{i}^{out}\left(t\right) & ={\vec{out}}_{i}\left(t\right)-{\vec{x}}_{i}\left(t\right) \end{array}$$

(7) $$\begin{array}{cc} {\vec{a}}_{i}\left(t\right) & =\sum_{j\in S_{n}}\frac{Q_{j}^{3}}{(|{\vec{x}}_{i}\left(t\right)-{\vec{x}}_{j}\left(t\right){|}^{3}\cdot T_{n}}\left({\vec{x}}_{i}\left(t\right)-{\vec{x}}_{j}\left(t\right)\right) \end{array}$$

(8) $$\begin{array}{cc} {\vec{v}}_{i}\left(t+1\right) & =0.5\cdot {\vec{v}}_{i}\left(t\right)+0.5\cdot {\vec{v}}_{i}^{in}\left(t\right)+0.5\cdot {\vec{v}}_{i}^{out}\left(t\right)+{\vec{a}}_{i}\left(t\right). \end{array}$$

Similar to Equation (4), $Q_{j}$ is a constant value and is used for the repulsion term in Equation (7). As either $\vec{in}$ or $\vec{out}$ will always be equal to ${\vec{x}}_{i}$, one of the velocity components will be 0. As such, the usually included scaling values $c_{1}$ and $c_{2}$ as well as the random variables $r_{1}$ and $r_{2}$ are set to a constant value 0.5 for all velocity components including the inertia weight. The reduced randomness helps the algorithm to consistently return to the object contour while the equal weight for the inertial velocity allows particles to overcome narrow corners and highly concave contours.

To prevent an immediate collapse to the position of the first particle, an increased but decaying repulsion factor between members of this sub-swarm is used to retain diversity in the sub-swarm, shown in Equation (7) with $T_{n}$ denoting the age of the sub-swarm, starting with 1.

### 5.4. Contour Trace Phase

The goal of this phase is to keep the particles as close as possible to the contour and create an archive *A* of the positions to reconstruct the complete contour. This phase starts only if during the Contour Search Phase, the distance of a particle to the contour is less than a threshold ϵ. More precisely, after the update of the ${\vec{in}}_{i}\left(t\right)$ and ${\vec{out}}_{i}\left(t\right)$ values, if their difference is less than a threshold ε, the contour tracing is triggered. In this phase, instead of using the repulsive force ${\vec{a}}_{i}\left(t\right)$, an additional term representing the direction of the edge of the contour is being included in the velocity update equation as follows:

(9) $$\begin{array}{cc} {\vec{e}}_{i}\left(t\right) & ={\vec{out}}_{i}\left(t\right)-{\vec{in}}_{i}\left(t\right) \end{array}$$

(10) $$\begin{array}{cc} {\vec{n}}_{i}\left(t\right) & =\left[\begin{array}{cc} 0 & -1\\ 1 & 0 \end{array}\right]\frac{{\vec{e}}_{i}\left(t\right)}{\left|\right|{\vec{e}}_{i}\left(t\right)\left|\right|} \end{array}$$

(11) $$\begin{array}{cc} {\vec{v}}_{i}\left(t+1\right) & =\frac{v_{i}\left(t\right)+\left({\vec{in}}_{i}\left(t\right)-{\vec{x}}_{i}\left(t\right)\right)+\left({\vec{out}}_{i}\left(t\right)-{\vec{x}}_{i}\left(t\right)\right)}{2}+s\;n_{i}\left(t\right). \end{array}$$

**Figure 2:**
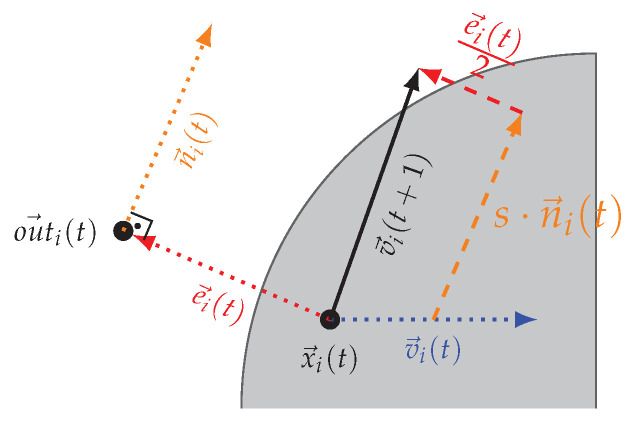
Contour Tracing Phase: Particle *i* is inside the object with velocity ${\vec{v}}_{i}\left(t\right)$ and ${\vec{e}}_{i}\left(t\right)$ = $\vec{out}{\;}_{i}\left(t\right)$. ${\vec{v}}_{i}\left(t+1\right)=0.5\left({\vec{v}}_{i}\left(t\right)+{\vec{e}}_{i}\left(t\right)\right)+{\vec{n}}_{i}\left(t\right)$.

With sufficient proximity of ${\vec{in}}_{i}\left(t\right)$ and $\vec{out_{i}\left(t\right)}$, the contour will be perpendicular to the vector ${\vec{e}}_{i}\left(t\right)={\vec{out}}_{i}\left(t\right)-{\vec{in}}_{(}t)$. This vector is either equal to ${\vec{in}}_{i}\left(t\right)$, in case the particle is currently outside of the contour or equal to ${\vec{out}}_{i}\left(t\right)$ if the particle is inside the contour. Using this approximation, the particle travels along one of the normal vectors of ${\vec{e}}_{i}\left(t\right)$, denoted ${\vec{n}}_{i}\left(t\right)$, as shown in Figure 2. The step size *s* defines the accuracy of the of the contour approximation as well as the time required to fully explore the contour. The smaller this value is, the higher the accuracy. The final step in the contour trace phase is to add the particle position to the archive *A*, which represents the contour approximated by the algorithm. This archive is in fact an approximation of the set *P* defined in Section 2. To reduce the size of the archive, a particle position is only added if the particle just left the contour, that is, the measured value fell below the detection threshold during the current iteration. This also helps increase the accuracy as the maximum error is thus bounded directly by the step size.

## 6. Evaluation

To evaluate the performance of our proposed algorithm, we have performed several experiments and compare the results with an state-of-the-art approach from the literature on image processing [19] which is an implementation of the approach proposed in Reference [14]. We use the result from this deterministic approach as a baseline and intend to find out, if our proposed PSCS can reach the optimal result. In order to measure and compare the quality of the results, we introduce an adapted version of the Inverted Generational Distance (IGD) indicator from the context of multi-objective optimisation [20]. Our modified the IGD indicator calculates the average of the distances between reference solutions (pixels) $p_{ref}$ and the closest solution $p_{A}$ stored in the archive *A*:

(12) $$\begin{array}{cc} IGD & =\frac{1}{\left|ref\right|}\sum_{\forall p_{ref}\in ref}\min_{p_{a}}\left|\right|p_{A}-p_{ref}\left|\right|, \end{array}$$

where ref is the set of reference points which are located on the contour and are computed using the state-of-the-art approach from the image processing literature [19]. In this way, all the reported IGD values contain the comparison with the exiting approach from the literature. The smaller the IGD values, the better is the quality of the obtained solutions. With this metric, we can additionally measure the quality of solutions for contours with various shapes including holes. Moreover, the scale of the image cannot influence the results.

**Figure 3:**
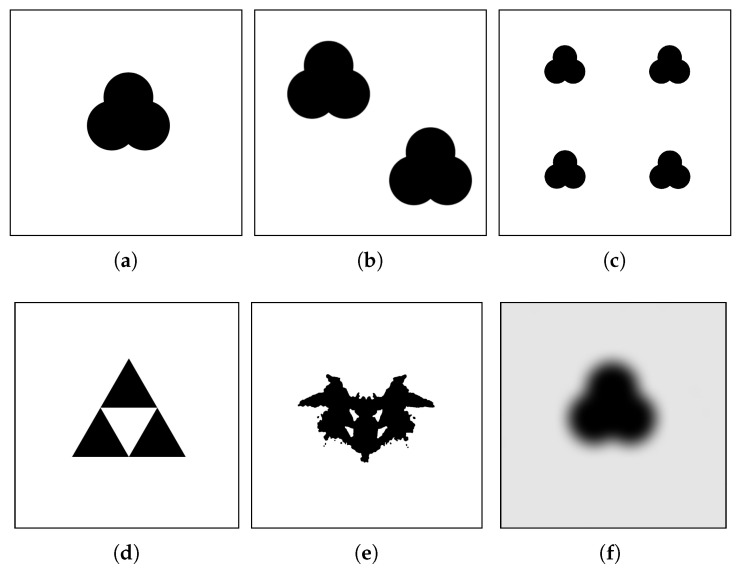
Test images. (**a**) Blot; (**b**) DoubleBlot; (**c**) QuadrupleBlot; (**d**) Sierpinski triangle; (**e**) Rorschach; (**f**) Blot Blur.

In addition to IGD, we study the convergence rate of PSCS over time. We measure how fast the outcome of the algorithm converges towards the real contour. To this end, the algorithm is evaluated on a variety of images containing various contours with varying parameter settings. To ensure statistical significance of the results, each experiment is performed with 31 independent runs and we set a maximum amount of 50,000 function evaluations. We have selected various test images with different shapes of objects as shown in Figure 3.

### 6.1. Parameter Setting

The proposed PSCS algorithm contains a variety of parameters, which have to be defined. Table 1 shows the selected parameters for the experiments. In addition, preliminary experiments have shown that ε, the threshold to enter the *contour trace phase* does not influence the result as long as the value is chosen large enough (ε>10). Therefore, we use a fixed value of ε=25 in all of the following experiments. The value of ε only influenced the robustness of the algorithm, calling for larger values in case errors are introduced. For analysing the impact of the initial positions of particles, three distinct distributions are implemented—line, point and random distribution. For the line distribution all particles’ initial positions are equally spaced out along one border of the search space. When set to point distribution, all particles start at the same randomly chosen point outside of the object. Random distribution assigns each particles on random starting positions outside of the contour. We take the repulsion factor $Q_{i}$ to be 16 as recommended by [17].

### 6.2. Experiments

**Figure 4:**
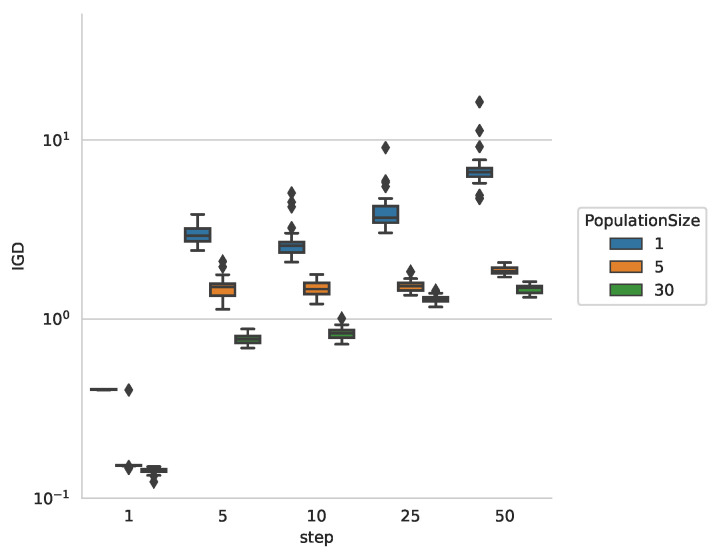
Inverted Generational Distance (IGD) for different step- and population sizes for Figure 3a.

In order to evaluate the influence of the parameters on the quality of the results from our proposed approach, we start the experiment on various parameter combination on the the blot image as shown in Figure 3a. Figure 4 shows the IGDvalue for all independent runs of the algorithm with different population sizes and step sizes *s*. We can observe that the step size has the largest impact on the measured IGD values. This concurs with our expectations as the step size directly influences the velocity of the particles during the contour trace phase. When the step size is smaller, the particle remains closer to the contour. In case the step size is larger, the particle may travel further from the contour in one time step resulting large IGD values.

Concerning population size, we observe that the larger values yield improved performance. Since the number of overall function evaluations is fixed, the improvement can not stem from more evaluations. Most likely the larger population sizes can help to obtain more diversity, thus improving the initial exploration and the overall result. Varying the sub-swarm size and initial particle distribution in this experiment does not result in significant improvement of the performance. This can be explained by the fact that the particles do not exchange information during the contour trace phase and therefore nothing is gained by a larger sub-swarm size.

In fact, the results for the first experiments are the best results we can expect, as there is an upper bound for how far the point in the archive can be from the contour (dependent on the step-size) and IGD value is within that boundary. This means that there are no areas in the contour that are not covered by the archive. In further experiments, we continue with the following parameters. The step size is set to 5 units and ε=25 and if not specifically indicated we take 6 subswarms with 5 particles each. In addition we use a random initialisation of particle positions. While a random initialisation is not necessarily the best setting for the above experiment, the random initialisation strategy performs better in the more complex settings, with more complex shapes and multiple objects in the image.

### 6.3. Experiments on Different Shapes

In the following experiments, we study the performance of our proposed approach on more complex images with multiple objects in the image, various scales and shapes (convex, concave and mixture).

**Figure 5:**
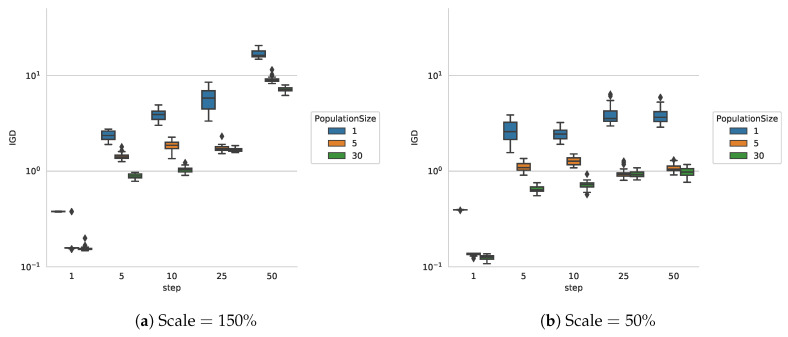
IGD for Figure 3a with different scaling.

**Different sizes of the object:** In order to investigate the impact of the size of the object and therefore the size of the contour, we have changes the size of the object in the image to smaller (50%) and larger (150%) than the object in Figure 3a. For larger sizes we expect the IGD indicator to increase, as more area needs to be covered by the same amount of particles and the amount of reference-points increases. The experimental results shown in Figure 5 confirm this expectation, although the effect is only really noticeable for the larger step sizes. The parameters used for this experiment were the ones recommended above, with the exception of the sub-swarm size, which was chosen to be 1.

**Figure 6:**
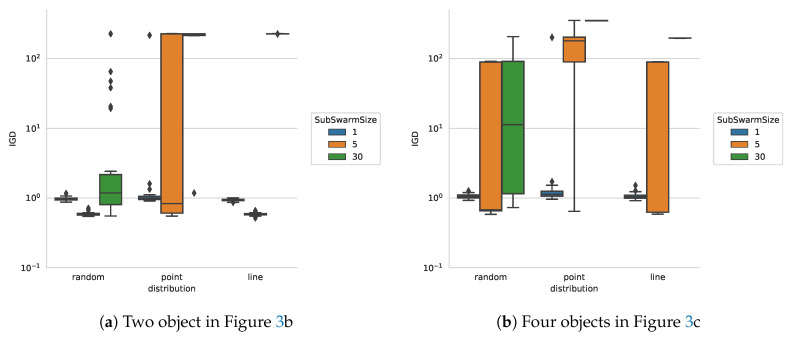
IGD for multiple objects in one search space.

**Multiple objects:** In the following experiments, we study the performance of our proposed approach in comparison with the results from the state-of-the-art [19] on images with several distinct objects. For this purpose, we create two images, one with two blots (Figure 3b) and the second with four blots (Figure 3c). Since the particles within one sub-swarm will never leave a contour after it is detected, we expect that the amount of sub-swarms and particles have a major impact on the performance. Figure 6 shows the IGD-performance of the algorithm with two and four objects.

With a population size of 30 particles a sub-swarm size of 30, all particles are merged into one sub-swarm, hence only one of the object can be detected for most distributions. In case some of the individuals pass the other contour during the initial convergence phase multiple contours can still be found. For this reason the initial distribution of the particles has a large impact on performance. The spread of the particles provided by a randomised distribution of starting position greatly increases the probability of all objects being detected, even if all particles are assigned to a single sub-swarm. For the line and point-based distributions, multiple objects are reliably detected only if the sub-swarm size is small enough and individual particles are not influenced by the detection of other objects.

**Figure 7:**
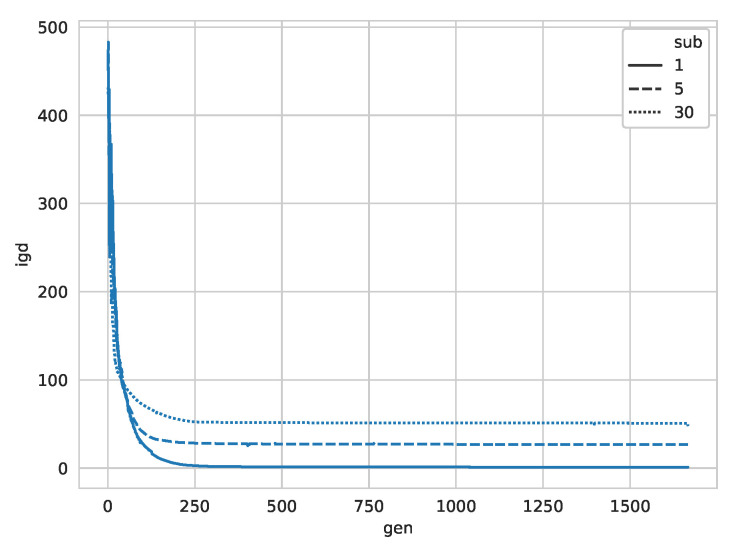
IGD over time for the QuadrupleBlot image in Figure 3c.

In the following we evaluate the convergence rate for the image with 4 objects as shown in Figure 7. The IGD values converge quickly to near its final value. Clearly visible is the different performance for the different sub-swarm sizes, with small, more numerous sub-swarms outperforming the larger sub-swarms. This quick convergence suggests that, after a while, the algorithm could be tuned to start another search for different contours, as little improvement in accuracy is gained after generation 200.

**Figure 8:**
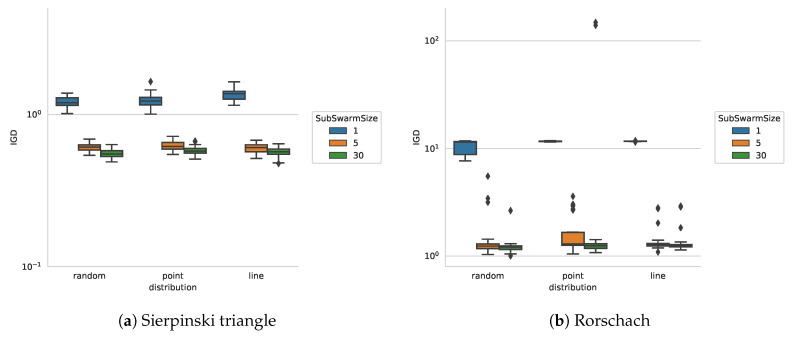
IGD for images in Figure 3d,e.

In the next step, we investigate the images containing objects with holes. As the algorithm is made to follow the contour of an object, objects with holes seem to pose a challenge. In the following we take two different images as shown in Figure 3d,e. The results of the IGD values shown in Figure 8 indicate that our approach obtains very good IGD values for Sierpinski triangle while its performance deteriorates for all the settings on the Rorschach image.

Interestingly, the random distribution of the initial population outperforms the line and point distribution for Rorschach images. We have observed that if the contour has a hole, only a sub-swarm of a size greater than one can locate this hole, since particles from the same sub-swarm occasionally exchange their memories. Sub-swarm size also plays a significant role when multiple distinct contours need to be found. The initial particle distribution is important for finding the contour in the beginning of an algorithm run. While it has a minor effect for the experiment on the blot image, as there is only one continuous contour, it plays a much important role for Rorschach images. Additionally, we found out that it is impossible for a single particle to find a hole unless the distance from the edge to the hole is smaller than the step size.

**Figure 9:**
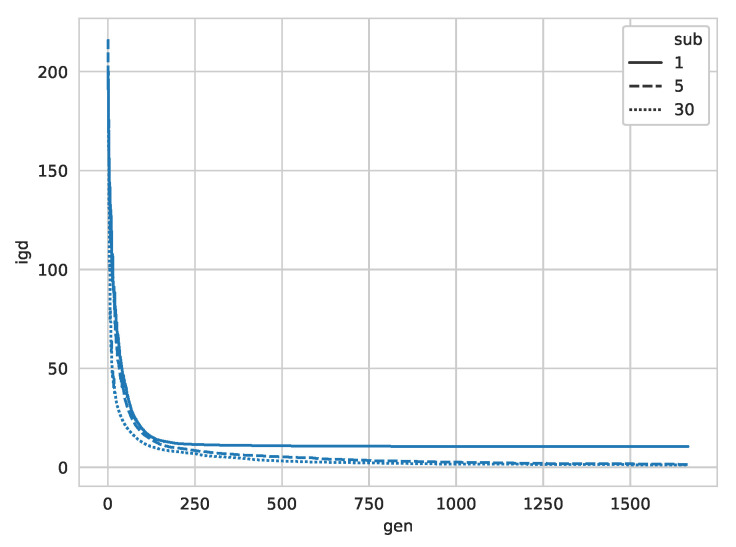
IGD over time for the Rorschach image.

When looking at the convergence rate for the Rorschach image, as shown in Figure 9, we observe that the algorithm quickly converges. However, the larger sub-swarm sizes continue to improve over several hundred generations. This is because the larger sub-swarm size is to find more holes in the image, which is consistent with the above results.

**Figure 10:**
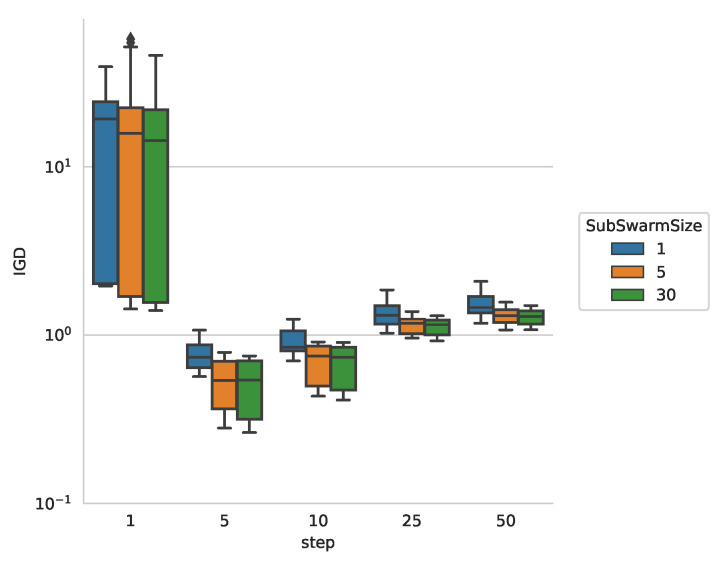
IGD for the blurred blot image in Figure 3f.

**Image with fuzzy contour:** So far we have tested PSCS on binary images. In the following experiments, we intend to study PSCS on images with fuzzy contours as shown in Figure 3f. To obtain a reference set for the IGD metric using the OpenCV contour search algorithm, which only works for binary images, we convert the image to a binary image using a threshold value of 127. This is the same value used for the threshold θ in the PSCS algorithm, which is executed on the grey-scale image. The results of IGD comparison in Figure 10 show that PSCS delivers a comparable result to those on the binary images, indicating that the algorithm is capable of performing accurately in non-binary search spaces.

## 7. Conclusions and Future Work

In this paper, the Particle Swarm Contour Search (PSCS) algorithm is presented. The algorithm can be used to find the contour of an object in a binary image. Using a PSO based algorithm for this purpose is rather unconventional, but since PSO is a very effective search mechanism, we aimed to investigate its performance for such problems. The main idea of our proposed PSCS algorithm is to find and follow the contour of an object. In the experiments, we compared our approach with a deterministic approach from the literature on image processing and used an adapted version of the IGD-indicator [20] to compare results. The experiments were performed and analysed on a set of benchmark images. Our experiments show that the step size (which defines the accuracy of the contour search) has the largest impact on the performance of the algorithm. While a small step size yields a more accurate estimate of the outline of he plateau, it also slows the process of covering the whole contour. Some of the current weaknesses of the algorithm could be addressed in future versions of the algorithm. First, the archiving strategy could be improved by pruning the archive. In addition, the algorithm could detect convergence and start new exploration once a contour is sufficiently covered. In some cases the particles can get stuck in a circular motion, this could be alleviated by introducing stochasticity in the particle movement. While not yet a perfect replacement for image based contour search, the work done shows a promising potential alternative for cases that render imaging technologies infeasible. In addition, we see possible applications of the algorithm as an extension of PSO algorithms that can tackle special fitness landscapes in Swarm Robotics.

## Figures and Tables

**Table 1:** Parameter values used for experiments.

Category	Parameter	Values
General	Population Size	1,5,30
	Sub-swarm Size	1,5,30
	initial distribution	Line, Point, Random
Contour search/trace phase	Step-size *s*	1,5,10,25,50
	Threshold ε	25

## Abbreviations

The following abbreviations are used in this manuscript:

PSCS	Particle Swarm Contour Search
PSO	Particle Swarm Optimisation
IGD	Inverted Generational Distance
